# Enhancing Functional and Visual Properties of Paulownia Wood Through Thermal Modification in a Steam Atmosphere

**DOI:** 10.3390/polym17152000

**Published:** 2025-07-22

**Authors:** Beata Doczekalska, Agata Stachowiak-Wencek, Krzysztof Bujnowicz, Maciej Sydor

**Affiliations:** 1 Department of Chemical Wood Technology, Faculty of Forestry and Wood Technology, Poznań University of Life Sciences, ul. Wojska Polskiego 38/42, 60-637 Poznań, Poland; agata.stachowiak@up.poznan.pl; 2 Institute of Natural Fibers & Medicinal Plants–National Research Institute, ul. Wojska Polskiego 71B, 60-630 Poznań, Poland; krzysztof.bujnowicz@iwnirz.pl; 3 Faculty of Engineering Management, Institute of Safety and Quality Engineering, Poznań University of Technology, 60-965 Poznań, Poland; maciej.sydor@put.poznan.pl

**Keywords:** paulownia wood, thermal modification, FTIR spectroscopy, color, compressive strength

## Abstract

*Paulownia elongata* wood is characterized by rapid mass gain, but its limited mechanical strength hinders engineering applications. This study aimed to determine the effect of thermal modification in a steam atmosphere (at temperatures of 180 °C and 190 °C for 12 or 6 h with 3 or 6 h of steam dosing) on wood’s selected physicochemical and aesthetic properties. Color changes (CIELAB), chemical composition (FTIR), density, and compressive strength parallel to the grain were evaluated. The results showed a clear darkening of the wood, a shift in hues towards red and yellow, and an increase in color saturation depending on the treatment parameters. FTIR spectroscopy confirmed a reduction in hydroxyl and carbonyl groups, indicating thermal degradation of hemicelluloses and extractives. Wood density remained relatively stable, despite observed mass losses and reduced swelling. The most significant increase in compressive strength, reaching 27%, was achieved after 6 h of modification at 180 °C with a concurrent 6 h steam dosing time. The obtained results confirm that thermal treatment can effectively improve the functional and visual properties of paulownia wood, favoring its broader application in the furniture and construction industries.

## 1. Introduction

In recent years, an increase in interest in the cultivation of *Paulownia*, a genus native to Asia, has been observed in Europe. These fast-growing plants are notable for their high adaptability to diverse climatic conditions [1]. The growing popularity of paulownia has contributed to its widespread introduction in many countries worldwide and has led to the development of numerous hybrid varieties. The genus paulownia (*Paulownia* spp.), comprising 17 species, is primarily cultivated for bioenergy purposes [2]. However, paulownia wood has gained attention as a lightweight construction material due to its low density and favorable insulating properties [1,3,4]. Its workability and ease of machining further enhance its appeal for a variety of applications—from the furniture industry to light structural elements [5]. Given its characteristics, paulownia wood holds significant potential as a sustainable alternative to expensive tropical hardwoods, such as balsa [6]. Despite these advantages, the naturally low density of paulownia wood often correlates with inferior mechanical performance when compared to conventional structural timber species [7]. Further research into its modification and reinforcement is warranted to expand its range of structural applications. Various types of wood modification offer the possibility to alter and improve its functional properties. One of the most commonly applied methods today is thermal modification, aimed at enhancing characteristics such as dimensional stability, hardness, biological resistance, and color [8,9,10]. This process involves the controlled heating of wood within a temperature range of 160 °C to 240 °C, typically in a steam atmosphere, leading to chemical alterations in the wood structure. Research on the thermal modification of different wood species has been conducted for several decades [11]. However, it is worth noting that thermal treatment, particularly at higher temperatures, often reduces mechanical strength, which remains one of the main limitations of this technology and has been widely observed across various wood species [12]. Previous studies on the thermal modification of paulownia wood have primarily aimed to improve its dimensional stability [13], biological resistance [14], and other functional properties. In a study by Pásztory et al. [15], changes in density and thermal conductivity of paulownia wood were assessed before and after thermal treatment at 180 °C, 200 °C, and 220 °C. Thermal modification at 180 °C did not significantly affect the thermal conductivity; however, a density reduction of 5.6% was recorded. Treatments at 200 °C and 220 °C resulted in a notable decrease in thermal conductivity, reaching values of 0.068 W/m·K and 0.064 W/m·K, respectively. The lowest thermal conductivity achieved through thermal modification was comparable to standard insulation materials and substantially lower than conventional softwood-based materials.

Kaygin et al. [16] examined the impact of thermal treatment at varying temperatures (160 °C, 180 °C, 200 °C) and durations (3, 5, 7 h) on the physical properties of *Paulownia elongata* wood. The study revealed that heat treatment reduced swelling, density, equilibrium moisture content, and lightness (L*), with the extent of changes increasing with temperature and duration. The most significant decreases were observed at 200 °C for 7 h, while the smallest changes occurred at 160 °C for 3 h. Zhang et al. investigated the effects of elevated temperatures (20–220 °C) and moisture content on the mechanical properties of Chinese paulownia wood under bending, tangential shear, and radial shear. The failure modes remained consistent despite increasing temperature [17]. Both strength and modulus of elasticity decreased with rising temperature and moisture, with the modulus of elasticity showing greater retention than the modulus of rupture. Peak deformations increased between 120 and 180 °C due to polymer softening. Shear specimens exhibited more brittle behavior than the pseudo-ductile failure observed in bending specimens. Esteves et al. demonstrated that thermal modification of *Paulownia tomentosa* wood at 212 °C leads to a decrease in the relative content of hemicelluloses, accompanied by an increase in other components such as α-cellulose, lignin, and extractable substances soluble in dichloromethane, ethanol, and water. Some original compounds disappeared in the heat-treated wood, while the amounts of compounds associated with lignin degradation, including vanillin, syringaldehyde, vanillic acid, syringic acid, conifer-aldehyde, and sinapaldehyde, appeared or increased. The treatment also caused a reduction in density, bending strength, and stiffness [18]. The dimensional stability improvement indices (anti-shrink or anti-swelling efficiency, ASE) were approximately 10% in the radial direction, 23% in the tangential direction, and 30% in the longitudinal direction. After three cycles, no significant deterioration in dimensional stability was observed, and a slight increase in ASE was even noted in the tangential direction. Water absorption decreased after thermal modification, with minor differences between cycles except for a slight increase after the third cycle. It was found that higher temperatures or more prolonged treatment durations could further improve dimensional stability but would also negatively affect the mechanical properties of the wood. In the study by Dogu et al., the microscale structural changes in *Paulownia tomentosa* wood subjected to thermal compression were analyzed. The wood samples, measuring 500 mm × 100 mm × 20 mm, were compressed under a pressure of 2 MPa in the tangential direction at temperatures of 150 °C and 170 °C for 45 min. Microscopic analyses revealed that the higher temperature (170 °C) caused slightly greater damage than 150 °C, and the distribution of deformations within the growth rings was uneven. No cell collapse was observed in the wood structure after thermal compression. The cited authors concluded that the shape of the cells and their arrangement in the growth rings along the loading direction were key factors influencing the uneven distribution of damage and the absence of cell collapse in the wood structure [19].

The literature review indicates that thermal modification of paulownia wood improves its functional properties, such as dimensional stability, hardness, and hydrophobicity. Although valued for its low density and favorable insulating properties, paulownia wood exhibits relatively low strength parameters compared to conventional structural timber species, which limits its use in structural applications. Thermal treatment—dependent on temperature and duration—leads to chemical and microstructural changes that enhance the wood’s functionality, often at the expense of its mechanical properties. Thermally modified paulownia wood shows potential as a lightweight, durable, and environmentally friendly building material, though further research is needed to optimize its mechanical and functional performance. This study aimed to investigate the effects of two thermal modification temperatures (180 °C and 190 °C) and two steam treatment durations (3 and 6 h) on the color and selected physicochemical properties of paulownia wood (*Paulownia elongata*). The results may contribute to a better understanding of the mechanisms occurring during the thermal treatment of this species and provide a basis for optimizing process parameters to improve the functional properties of the wood, with consideration for its potential applications in the furniture industry, construction, and as an insulating material.

## 2. Materials and Methods

### 2.1. Paulownia Wood

The study employed *Paulownia elongata* wood sourced from Shandong Province, China. Before the experimental procedures, the lumber was cut into 20 mm × 20 mm × 30 mm specimens. Only defect-free samples, free from knots, cracks, and other visible imperfections, were selected to ensure material homogeneity and repeatability of the results. Six sample series, each consisting of 30 specimens, were prepared. Before each thermal modification process, the specimens were dried in a laboratory oven at 105 °C until a constant mass was achieved.

### 2.2. Methods

#### 2.2.1. Chemical Composition of Paulownia Wood

The specific components of paulownia wood (expressed as dry matter, DM) were analyzed following TAPPI standard methods. The procedures included: (a) holocellulose content determined using sodium chlorite (TAPPI T 9 wd-75); (b) determination of cellulose content using Seifert’s method with acetylacetone–dioxane mixture [20]; (c) pentosans content measured by Tollens’ method with phloroglucinol reagent (TAPPI T 233 cm-84); (d) lignin content determined via the TAPPI method employing concentrated sulfuric acid (TAPPI T 222 om-06); (e) substances soluble in organic solvents according TAPPI-T204 cm-0; (f) ash content measured according to TAPPI T 211 cm-86. The theoretical hemicelluloses content was calculated as the difference between holocellulose and cellulose. All values are averages calculated from five replicates (*n* = 5).

#### 2.2.2. Thermal Modification of Paulownia Wood

**Figure 1:**
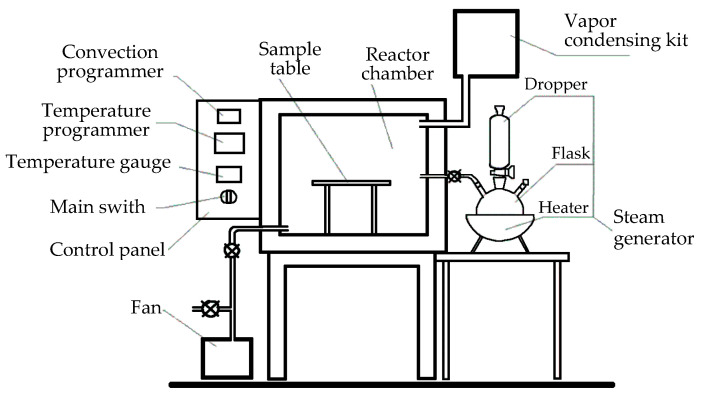
Schematic diagram of the thermal modification chamber.

The modification processes were conducted within a gas-tight laboratory chamber (Figure 1), which featured designated ports to supply steam and to remove gaseous reaction products. This chamber was intricately linked to a laboratory steam generator and a cooling system to manage uncondensed products, and the variants of wood modification applied in the process are presented in Table 1.

Samples after modification were stored in a desiccator at a controlled room temperature (20 ± 2 °C).

The efficacy of the wood modification process was evaluated by determining the weight percentage gain (*WPG*, %) and the bulking coefficient (*BC*, %) as described by Hill [12]. The changes in mass and volume were quantified using the formulas provided in Equations (1) and (2):

(1) $$WPG=\frac{M_{m}-\;M_{0}}{M_{0}}\times 100$$

(2) $$BC=\frac{V_{m}-V_{0}}{V_{0}}\times 100$$

where *M*_0_ is the mass of completely dry unmodified wood (g), *M_m_* is the mass of completely dry modified wood (g), *V*_0_ is the volume of unmodified wood sample (cm^3^), and *V_m_* is the volume of modified wood sample (cm^3^).

The density of wood samples was determined following the ISO 13061-2 standard [21]. Rectangular specimens were measured along three orthogonal directions using a caliper with a precision of 0.01 mm. Subsequently, the samples were weighed using a laboratory balance with an accuracy of 0.001 g. Density was calculated as the ratio of mass to volume.

#### 2.2.3. Fourier Transform Infrared Spectroscopy (FTIR) Measurement

Following the modification process, the control samples were analyzed using an Alpha FTIR spectrophotometer (Brüker Optics GmbH, Ettlingen, Germany), equipped with an attenuated total reflectance (ATR) accessory featuring a germanium crystal. Spectral data were collected within the 4000–600 cm^−1^ range, with a resolution of 4 cm^−1^. Each spectrum was obtained as the average of 32 consecutive scans to enhance accuracy and reduce measurement error. For each sample, three independent measurements were conducted.

#### 2.2.4. Color Measurements

Color measurements were conducted on the radial surfaces of both control and thermally modified samples. The color coordinates L*, a*, and b*—as defined by the Commission Internationale de l’Éclairage (CIE)—were recorded using a dual-beam d/8° spectrophotometer (mod. Datacolor 600, Lorentzen & Wettre, Kista, Sweden). Measurements employed the D65 standard illuminant and covered a spectral range of 360–700 nm. The spectrophotometer featured a maximum reflectance deviation of 0.15 and an average of 0.08. The sensor head had a diameter of 10 mm. Three specimens were analyzed for each thermal modification variant, with color measurements taken at three fixed points on each sample. The device was calibrated using a white calibration plate, a green reference plate, and a black trap.

The data presented in this study represent the mean values derived from nine replicate measurements. The color space defined by the L*, a* and b* axes constitutes a tristimulus system that quantitatively describes color coordinates. The total color change in the CIELAB-color-system was calculated according to Equation (3):

(3) $$∆E*=\sqrt{{\left(∆L*\right)}^{2}+{\left(∆a*\right)}^{2}+{\left(∆b*\right)}^{2}}$$

where ΔE* is the total color change, L* is the achromatic coordinate, or luminosity, with values ranging from 0 (black) to 100 (white) and a* and b* are the chromatic coordinates: Axis a* depicts green (a* < 0) or red (a* > 0) color, and axis b* depicts blue (b* < 0) or yellow (b* > 0) color. The c* parameter expresses chroma and determines the ‘color purity’, whereas h is the color angle (0° represents red color on +a* axis, 90° represents yellow on +b* axis, 180° represents green on −a* axis, and 270° represents blue on −b* axis). The parameters ΔL*, Δa*, and Δb* express the difference in lightness, red–green, and yellow–blue components, respectively. The Δc* expresses the difference in color saturation, and Δ*h* defines the difference in hue.

#### 2.2.5. Compressive Strength Testing

The compressive strength parallel to the fibers was determined following the ISO 13061-17 standard [22], which specifies the method for measuring the ultimate stress in compression for small clear wood samples. Rectangular samples were prepared with dimensions of 20 mm × 20 mm × 30 mm (radial × tangential × longitudinal), ensuring that the load was applied along the longitudinal axis, parallel to the wood fibers.

Before testing, all specimens were conditioned to a constant mass in an atmosphere with a relative humidity of 65 ± 5% and a temperature of 20 ± 2 °C. The compression test was carried out using a universal testing machine (UTM) (mod. Inspekt Table 50, Hegewald & Peschke MPT, Nossen, Germany). The load was applied at a constant deformation rate, not exceeding 1.0 mm/min, until failure occurred. The maximum force recorded during the test was used to calculate the ultimate compressive stress, using the Equation (4):

(4) $$\sigma =\frac{F_{max}}{a\cdot b}$$

where *σ* is the ultimate compressive stress (MPa), *F*_max_ is the maximum load (N), *a*, and *b* are the dimensions of the specimen’s rectangular cross-section (mm).

Ten replicates (*n* = 10) were tested for each modification variant. Descriptive statistics, including mean values and standard deviations, were calculated to assess the variability and compare treatment effects.

#### 2.2.6. Statistical Analysis

A one-factor analysis of variance with repeated measures and a Bonferroni post hoc test was used to analyze *WPG* values. To analyze the ultimate stress in compression parallel to the grain between series, the following statistical methods were used: one-way ANOVA, Bonferroni post hoc test, and two-way ANOVA. The statistical analyses, including graph preparation, were performed using MS Excel using the built-in Analysis ToolPak add-in (release 2103, 13 April 2021, Microsoft Corp., Redmond, WA, USA).

## 3. Results and Discussion

### 3.1. Chemical Composition Analysis of Paulownia Wood

Table 2 summarizes the mean values and standard deviations of the chemical components of paulownia wood.

Holocellulose accounted for the most significant proportion of the wood’s chemical composition, reaching an average content of 78%. This high fraction level indicates a substantial presence of structural carbohydrates—cellulose and hemicelluloses—characteristic of softwood that grows rapidly. The average cellulose content was 43%, further highlighting the potential of this species for industrial applications.

The average lignin content was approximately 25%, corresponding to values typical for many hardwood species. Lignin, as a structural compound responsible for cell wall rigidity, plays a key role in shaping the mechanical strength of wood.

The extractive fraction content was approximately 11.5%. This level indicates the presence of bioactive compounds, such as tannins, flavonoids, and phenolic acids, which may play a significant role in the natural resistance of paulownia wood to biotic factors such as fungi and insects. The mineral content was low, at around 0.7%, which is typical for wood.

Literature data indicate that the chemical composition of Paulownia wood varies depending on the variety and hybrid. For *Paulownia elongata*, Ates et al. [23] reported a holocellulose content of 75.74%, cellulose—43.61%, and lignin—20.5%. They also determined the content of extractives soluble in an ethanol/benzene mixture at 3.76% and ash at 0.21%. The chemical composition obtained by these authors is similar to the results obtained in the present study, especially concerning holocellulose and cellulose content, which confirms the repeatability of the chemical characteristics of this wood species.

The wood of *Paulownia tomentosa* was characterized by a cellulose content of 51%, hemicelluloses of 30%, lignin of 23.5%, and extractives of 11.8% [24]. Studies on the dihybrid *P. elongata* × *fortunei* showed that the wood’s main component was glucose (39.7%), originating primarily from cellulose. Hemicelluloses (xylan, galactan, and mannan) accounted for 14.7%, lignin—21.9%, and extractives—5.6%. The ash content was 0.5% [25]. In the case of *Paulownia COTEVISA-2* wood, cellulose accounted for 48.15%, lignin—27.48%, and pentosans—7.38%. The content of ethanol-soluble substances was 5.51%, and ash was 0.4% [26].

In summary, paulownia wood exhibits a high content of carbohydrate fractions (cellulose and hemicelluloses), a moderate level of lignin, a low mineral content, and a variable content of extractive substances.

### 3.2. Determination of Weight Percentage Gain, Density, and Bulking Coefficient

The weight percentage gain (*WPG*, %), densities (g/cm^3^), and changes in the swelling ratio (bulking coefficient, *BC*, %) were evaluated for six batches of samples subjected to thermal modification (see Table 1). Each measurement was performed in ten replicates (*n* = 10). Descriptive statistics summarizing the results are presented in Table 3.

Based on the obtained results, the thermal modification of paulownia wood does not significantly affect its density. The mean density values for all treatment variants ranged narrowly from 0.24 to 0.26 g/cm^3^, comparable to the control sample (0.26 ± 0.04 g/cm^3^). The lowest mean density was recorded for the 190/12/6 and 190/6/3 variants (0.24 ± 0.03, and 0.24–0.04, respectively). However, these differences fall within the standard deviation range and do not indicate an apparent effect of temperature or treatment duration on the material’s density. Therefore, it may be assumed that the thermal modification parameters applied in this study do not significantly impact the density of the wood.

It was observed that thermal modification of paulownia wood reduces mass and decreases the swelling ratio. Concurrently, the values of the weight percent gain (*WPG*) and bulking coefficient (*BC*) indicators became progressively more negative as the modification temperature increased. Furthermore, shortening the steam treatment time from 6 to 3 h results in more pronounced changes in both parameters.

A one-way repeated measures ANOVA revealed statistically significant differences between the treatment variants, F = 63.11, *p* < 0.001. Consequently, the null hypothesis was rejected.

**Figure 2:**
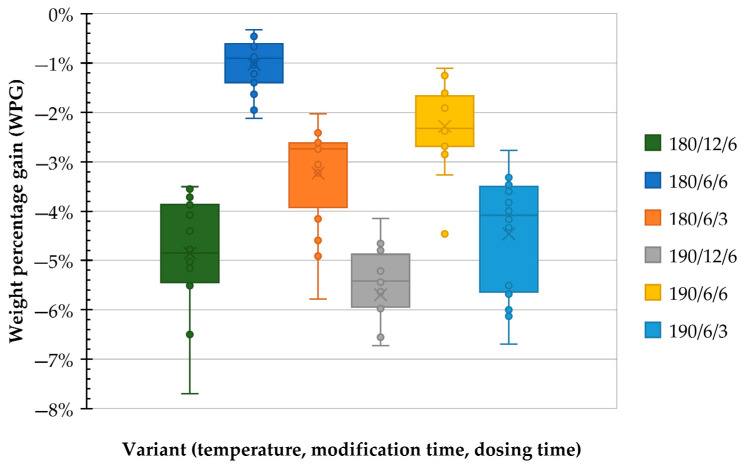
Weight percentage gain across variants, two clusters were marked.

The Bonferroni post hoc test identified two distinct clusters. The first cluster included the 180/6/6 and 190/6/6 variants, while the second cluster comprised the remaining treatment conditions: 190/12/6, 180/6/3, 190/6/3, and 180/12/6. These groupings are visible in Figure 2.

The greater variability in *WPG* values at 180 °C (particularly in the 180/12/6 and 180/6/3 variants) may result from the natural heterogeneity of *Paulownia elongata* wood, including anatomical and chemical variability, especially in hemicellulose and extractive content [19,27,28]. At lower temperatures, the modification process is more sensitive to minor fluctuations in parameters [16], and differences in treatment duration and the heating/cooling phases may further increase the variation in results [29].

According to the literature, paulownia wood is characterized by a relatively low initial density and stability of this parameter during thermal treatment at temperatures up to 180 °C. Despite minimal changes in density, a noticeable mass loss is observed starting from 180 °C, which distinguishes paulownia from other wood species [30]. The mass loss may be associated with the high content of hemicelluloses and extractives (see Table 2), which are more susceptible to degradation at lower temperatures. The chemical composition of paulownia wood—particularly the presence of extractives and the degradation of hemicelluloses—plays a key role in its behavior during thermal modification, contributing to the reduction in mass while maintaining relative density stability [31,32,33].

### 3.3. Fourier Transform Infrared Spectroscopy

**Figure 3:**
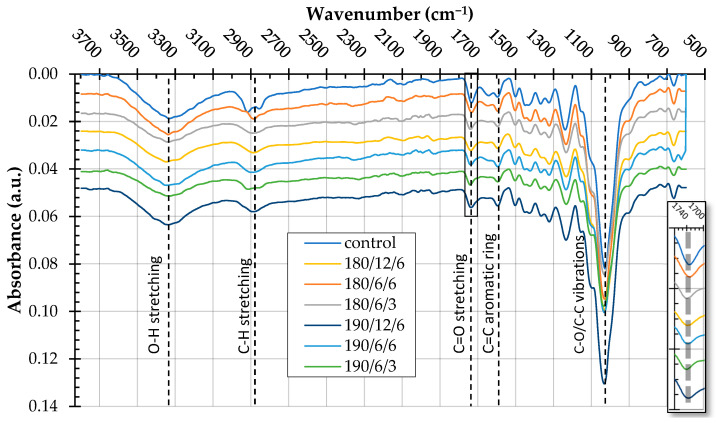
FTIR spectra of thermally treated paulownia wood samples.

Fourier transform infrared (FTIR) spectroscopy assessed the chemical changes occurring in paulownia wood due to thermal treatment (Figure 3). The recorded spectra revealed the presence of characteristic absorption bands associated with the main lignocellulosic components of wood, such as cellulose, hemicelluloses, and lignin, and indicated their variability depending on the temperature and duration of the treatment.

The assignment of characteristic absorption bands for paulownia wood is presented in Table 4.

In the region around ~3400 cm^−1^, broadband corresponding to the stretching vibrations of hydroxyl groups O–H present in cellulose, hemicelluloses, and lignin’s structure was identified. The control sample exhibited the highest band intensity at 3337 cm^−1^ compared to the thermally treated samples. The observed decrease in the intensity of this band may indicate a partial loss of –OH groups due to dehydration and condensation processes.

In the 3000–2800 cm^−1^ region, bands corresponding to the stretching vibrations of C–H bonds, characteristic of aliphatic fragments of cellulose and lignin, were identified. Shifts in peak positions and a decrease in intensity (with the lowest values observed in samples 180/6/6 and 180/6/3) may indicate the breaking of hydrocarbon chains in the wood polymer structure under the influence of temperature.

The presence of acetyl, carbonyl, and carboxyl groups (C=O), originating from oxidized hemicelluloses or lignin degradation products, was confirmed in the 1750–1700 cm^−1^ region. The control sample exhibited an absorption maximum at 1733 cm^−1^, while in the thermally modified samples, slight shifts and decreases in the intensity of this band were observed, which may indicate the breakdown of ester groups or oxidative restructuring of the polymer structure (Figure 3). Deacetylation of hemicelluloses constitutes one of the initial stages of thermal degradation of wood.

The band around 1595 cm^−1^, corresponding to the vibrations of C=C bonds in the aromatic structure of lignin, remained stable in terms of position, while its intensity decreased with increasing temperature and treatment time, which may indicate partial degradation or condensation of lignin.

In the so-called fingerprint region (1200–1000 cm^−1^), which primarily corresponds to stretching vibrations of C–O and O–H bonds in the structure of cellulose and hemicelluloses, distinct differences in band intensities were observed between the control sample and the modified samples. The increased signal intensity in sample 190/12/6 may indicate structural transformations related to hemicellulose degradation and changes in the degree of ordering of the polysaccharide chains.

**Figure 4:**
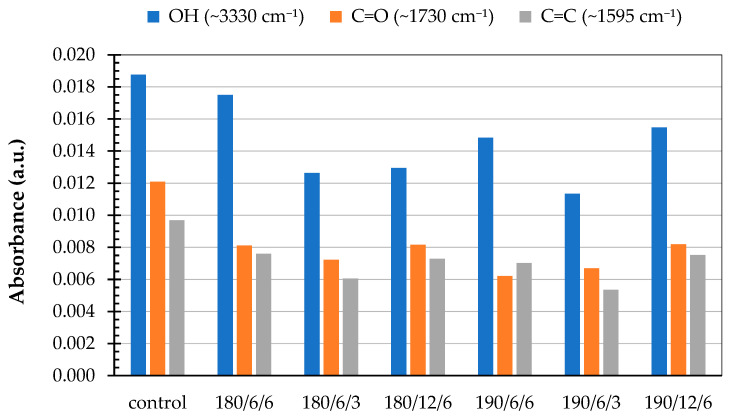
Changes in the intensity of functional group bands –OH (~3330 cm^−1^), C=O (~1730 cm^−1^), and C=C (~1595 cm^−1^) in the FTIR spectra of paulownia wood before and after thermal modification.

Changes in the intensity of functional group bands –OH (~3330 cm^−1^), C=O (~1730 cm^−1^), and C=C (~1595 cm^−1^) in the FTIR spectra of paulownia wood before and after thermal modification are presented in Figure 4.

Comparative analysis of these three absorption bands confirmed that thermal treatment decreases their intensity compared to the control sample. The reduced intensity of the band at ~1730 cm^−1^ may indicate degradation of carbonyl groups associated with hemicelluloses. Their breakdown results in fewer hydrophilic groups and lower moisture absorption. Reduced moisture content limits swelling and decreases the risk of microcracking, contributing to improved mechanical strength. As Esteves et al. [18] revealed, moderate temperatures (around 180 °C) induce softening and reorientation of lignin within the cell walls. A decrease in the band’s intensity at ~1595 cm^−1^, corresponding to aromatic C=C bonds in lignin, may indicate condensation or partial redistribution of lignin. Under moderate heating conditions, new chemical bonds may also form between fragments of degraded hemicelluloses and lignin (e.g., methyl or ester bridges), resulting in increased structural stiffness of the wood [32].

The differences between the spectra of unmodified wood and thermally treated wood are difficult to interpret unambiguously due to the simultaneous occurrence of multiple chemical reactions. Nevertheless, slight changes in the FTIR spectra were observed even under the mildest treatment conditions, corresponding to small mass losses (Table 3). The subtle differences observed in the FTIR spectra may be associated with increasing mass loss resulting from progressive changes in the wood components. The reduction in the intensity of O–H and C–H bands, as well as the shifts and weakening of carbonyl and aromatic bands, indicate that dehydration, oxidation, and thermal degradation of the main lignocellulosic components of paulownia wood occur during thermal modification. The structural components of wood exhibit varying thermal resistance, which can be ranked as lignin > cellulose > hemicelluloses [12]. The thermal degradation of hemicelluloses, as observed in the FTIR spectra, is manifested by a decrease in the band’s intensity at approximately 1730 cm^−1^. As the modification temperature increases, a gradual reduction in intensity is also observed in the 1050–1030 cm^−1^ range, corresponding to C–O vibrations in the glucopyranose rings of cellulose and hemicelluloses. In turn, extractives present in paulownia wood at 11.5% (Table 2) are degraded mainly at the modification temperatures applied in this study, i.e., 180 °C and 190 °C.

### 3.4. Color Measurements

**Figure 5:**
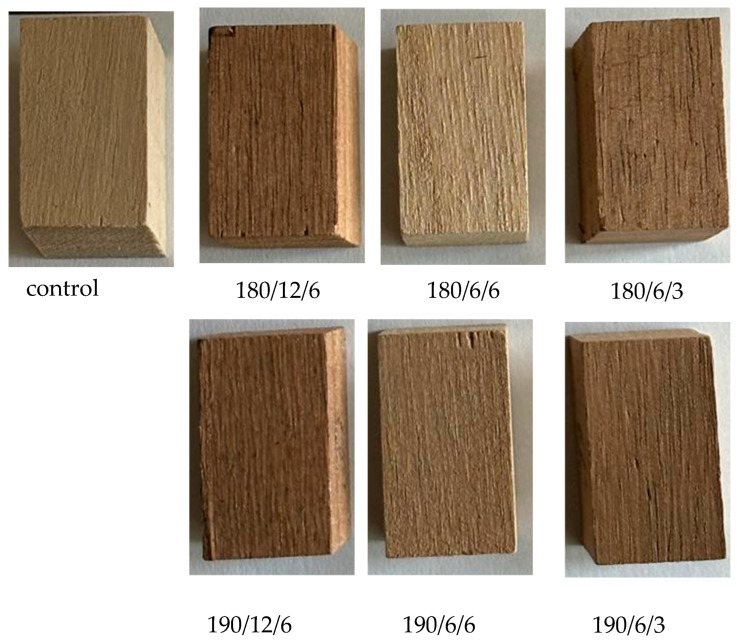
Paulownia wood samples before and after thermal treatment.

Figure 5 presents images of paulownia wood specimens taken before and after the modification processes.

As a result of the thermal modification of paulownia wood, significant changes were observed in the color parameters expressed within the CIELAB color system (Table 5).

The most noticeable effect was a systematic decrease in the thermally modified wood’s lightness coefficient (L*) compared to the control samples. The recorded changes indicate progressive darkening of the wood with increasing modification temperature, from 77.58 in the control sample to 43.94 units for the sample subjected to the most intensive treatment conditions (190/12/6).

Simultaneously, the a* value increased from 4.11 units to a maximum of 9.13 units, indicating a shift in color toward red hues. The b* coefficient, which reflects changes toward yellow shades, initially increased, reaching values above 21 units under the 190 °C for 6 h modification conditions (190/6/6). However, under prolonged treatment at the same temperature (190/12/6), it decreased to a value comparable to that of the control sample.

The chroma parameter (c*) also increased, indicating greater color saturation. However, for the sample modified under the highest temperature and most extended duration (190/12/6), a decrease in c* was observed, suggesting a possible loss in color intensity compared to samples modified under milder conditions.

The hue angle (*h*) decreased from 76.54 to 63.86, confirming the wood color tone shift toward redder and less yellow hues.

It is worth noting that shortening the steam dosing time from 6 to 3 h resulted in less pronounced changes in the color coefficients.

In summary, the thermal modification of paulownia wood led to a distinct change in color toward darker, warmer, and more saturated tones. The extent of these changes was dependent on both the temperature and the duration of the treatment.

The changes in color coordinates, expressed as differences (Δ) relative to the control sample and encompassing the components of the CIE Lab* color space (Δ*L*, Δ*a*, Δ*b*), as well as the total color difference (Δ*E*), indicate a significant influence of temperature and duration of thermal treatment on the color parameters of paulownia wood (Table 6).

The ΔL* values were negative across all modification variants, indicating a general darkening of the wood due to high-temperature exposure. The most significant reduction in lightness was observed for the sample treated at 190 °C for 12 h (190/12/6), with a ΔL* of –33.71 units. This result suggests that the darkening intensity increases with higher temperatures and prolonged steam exposure.

The Δa* and Δb* parameters, corresponding to shifts along the red–green and yellow–blue axes, respectively, exhibited varied trends. For Δa*, a consistent increase was observed, confirming a shift in wood color towards red hues following thermal modification. In the case of Δb*, an apparent increase was recorded at lower treatment temperatures, with the highest value observed for the 180/6/3 variant (Δb* = 5.62 units). However, under the most intensive conditions (190/12/6), the Δb* value significantly decreased to 1.22 units, which may indicate the degradation of chromophoric groups responsible for the yellowish tone of the wood.

Changes in chroma (Δc*) generally followed an increasing trend, indicating enhanced color saturation after thermal treatment. The highest Δc* value was recorded for the 180/6/3 variant (Δc* = 6.71 units). Conversely, changes in hue angle (Δ*h*) also showed an upward trend with increasing temperature and duration of treatment, suggesting a deepening of the wood’s color tone as the modification intensity increased.

The total color change, expressed by the Δ*E* parameter, also increased with the intensification of the thermal modification conditions. Δ*E* values ranged from 14.96 to 34.10 units. The highest color change (Δ*E* = 34.10) was observed in samples treated at 190 °C for 12 h with steam application for 6 h (190/12/6). These results indicate pronounced color differences between the modified and control samples, confirming the significant impact of thermal treatment parameters on the visual properties of paulownia wood.

Kim et al. [30] investigated the effect of thermal modification on the properties of royal paulownia wood (*Paulownia tomentosa*) and compared them with those of Suwon silver poplar (*Populus tomentiglandulosa*) and Korean red pine (*Pinus densiflora*). Samples from all three species were subjected to heat treatment at 160 °C, 180 °C, 200 °C, and 220 °C in an electric furnace for 2 h. Changes in color parameters, density, and mass loss were evaluated before and after the treatment. A marked decrease in lightness (L*) was observed in all species beginning at 200 °C. With increasing temperature, no significant changes were detected in the red-green (a*) or yellow-blue (b*) chromaticity values for royal paulownia and poplar [30].

However, in the Republic of Korea red pine, the b* value decreased sharply at 200 °C. Royal paulownia exhibited a noticeable total color change (Δ*E*) beginning at 180 °C, whereas poplar and pine displayed significant color changes from as low as 160 °C. In general, color differences across all species increased substantially with rising temperatures. Among the three species, mass loss due to heat treatment was highest in royal paulownia and lowest in pine. The mass loss showed an upward trend with increasing temperature, while density declined slightly. These results indicate that royal paulownia responds differently to heat treatment compared to the other two species, particularly in terms of the onset temperature of color change and extent of weight loss.

The obtained results confirm that thermal modification constitutes a practical and durable method for altering the color of paulownia wood. It can be successfully applied in industrial practice as an alternative to traditional coloring techniques, such as chemical dyes, stains, or varnishes. Furthermore, the findings indicate that by appropriately selecting the modification process parameters, it is possible to control the extent and intensity of color changes, thereby enabling precise tailoring of the aesthetic properties of paulownia wood following functional and design requirements.

### 3.5. Compressive Strength Parallel to the Fibers

The ultimate compressive stress parallel to the fibers was determined by testing seven series of samples, with ten replicates (*n* = 10) performed for each series. Descriptive statistics summarizing these results are presented in Table 7.

The results presented in Table 7 indicate that thermal modification positively affects the compressive strength of paulownia wood parallel to the grain. The average ultimate stress for the control sample was 21.2 MPa, while all thermally treated samples showed an increase in this value. The highest average strength, 26.93 MPa, was recorded for the 180/6/6 modification, representing an increase of approximately 27% compared to the unmodified wood. Other variants also demonstrated improved mechanical properties, with average values ranging from 24.16 to 25.47 MPa, corresponding to an increase of approximately 14–21%. This upward trend suggests that changes in the chemical structure of paulownia wood induced by exposure to temperatures of 180 °C and 190 °C—confirmed by infrared spectroscopy analysis (Section 3.3)—contribute to enhancing its compressive strength.

**Figure 6:**
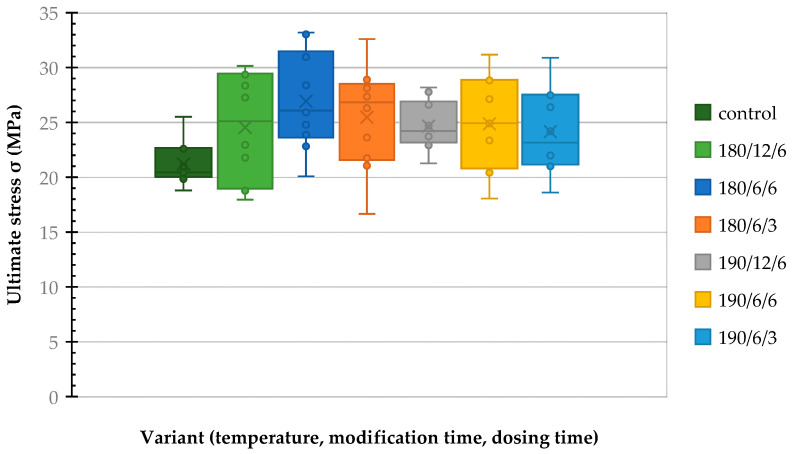
The average compressive strength of paulownia wood after thermal modification at different variants and durations, compared to the control sample.

Table 7 shows the average compressive strength increase after each thermo-modification variant and dosage time compared to the control sample. These data are presented graphically in the Figure 6.

To evaluate the impact of thermal modification on the compressive strength of paulownia wood along the grain, one-way and two-way analyses of variance (ANOVA) were performed within the statistical analysis framework. The one-way analysis of variance aimed to determine whether there are statistically significant differences in the mean compressive strength between the control group and the thermally modified wood samples. The statistical hypotheses were formulated as follows:

- Null hypothesis (H_0_): There is no significant difference in the mean compressive strength between the experimental and control groups.
- Alternative hypothesis (H_1_): There is a significant difference in the mean compressive strength between at least one experimental group and the control group.

The one-way ANOVA results indicated no statistically significant differences between the compared groups (*F* = 1.92; *p* = 0.0912). Consequently, the null hypothesis could not be rejected at the conventional significance level (α = 0.05), suggesting that the observed differences in compressive strength among the groups may be due to random variation rather than the effect of thermal treatment.

Despite the overall non-significant result of the ANOVA, a Bonferroni post hoc test revealed a statistically significant difference between the control group and the group modified under the 180 °C/6 h/6 h condition (*p* < 0.05). The mean compressive strength for these two groups was 21.2 MPa and 26.93 MPa, respectively. This finding suggests that, based on the available data, this specific thermal treatment may have led to a statistically significant improvement in compressive strength.

A two-way ANOVA was performed to assess the influence of two factors on strength. The selected factors were thermo-modification temperature (180 and 190 °C) and steam dosing time (3 and 6 h). The results of the two-way ANOVA are presented in Table 8.

At the significance level of α = 0.05, none of the analyzed factors (temperature, time, or their interaction) showed a statistically significant effect on the dependent variable, i.e., compressive strength.

Based on the conducted statistical analyses, no significant effect of modification temperature, dosing time, or their interaction on the compressive strength of paulownia wood was found. An exception was observed in the pairwise comparison between the control group and the 180/6/6 variant, demonstrating a significant improvement in mechanical properties.

In recent years, more studies have indicated that moderate thermal treatment of wood can improve its mechanical properties, including compressive strength. Suri et al. [40] demonstrated that paulownia tomentosa wood modified at 180 °C exhibited higher compressive strength than control samples, although this effect diminished at higher temperatures. Nocetti et al. [41], studying paulownia wood from northern Italy, found that thermal modification may enhance biological durability and dimensional stability, despite a slight decrease in overall mechanical strength. Park et al. [42] confirmed that heat treatment causes degradation of hemicelluloses and reduces the sorption capacity of wood, which is associated with mass loss and decreased equilibrium moisture content. These effects directly translate into improved dimensional stability of the material. Moreover, Mandraveli et al. [43] pointed out that increases in strength and hardness after thermal modification may result from lignin condensation and migration, filling microscopic voids in the cell walls. Similarly, Nakagawa et al. [44] observed an increase in the modulus of elasticity of western fir wood after modification at 167 °C.

In light of these findings, the increase in compressive strength observed in this study can be interpreted as a synergistic effect of several mechanisms: degradation of hemicelluloses (leading to reduced hygroscopicity), redistribution and condensation of lignin (enhancing the structural integrity of cell walls), and possible formation of cross-linked bonds under steam-assisted thermal modification conditions.

## 4. Conclusions

The thermal modification of *Paulownia elongata* wood in a steam atmosphere, applied for 3 or 6 h at temperatures of 180 °C and 190 °C, resulted in distinct color changes of wood, confirmed by measurements of color parameter changes in the CIELAB color space. A darkening of the wood was observed, along with a shift in its hue towards warmer, more saturated tones, particularly red and yellow. The total color difference (Δ*E*) ranged from 15 to as much as 34 units. FTIR analysis revealed a reduction in hydroxyl and carbonyl groups, indicating thermal degradation of hemicelluloses and extractives. These changes were dependent on the thermomodification process parameters, i.e., temperature, treatment time, and steam dosing duration, which allow for shaping the visual effect of the wood.

The heat treatment also resulted in a noticeable weight loss (*WPG* to −5.7%) and a reduction in the swelling ratio (*BC* to −1.5%), while maintaining a relatively stable density (0.24–0.26 g/cm^3^). These effects become more pronounced at higher temperatures and shorter steam treatment times.

A significant observed effect of the thermo-modification process was an increase in the compressive strength of the wood parallel to the grain—even by 27% compared to unmodified wood. The best results were achieved at 180 °C with a steam dosing time of 6 h. The observed changes result from complex chemical processes occurring in wood structure, primarily the thermolysis of hemicelluloses and extractives, which influence the physicochemical properties of paulownia wood.

Thermal modification can, therefore, be an effective method to improve the strength and aesthetics of paulownia wood without significantly deteriorating its mechanical and physical properties. This allows for expanding paulownia’s applications in the furniture and construction industries.

## Figures and Tables

**Table 1:** Thermal modification parameters of paulownia wood.

Modification Variant	Modification Temperature (°C)	Steam Dosing Time (h)	Steam Exposure Time (h)
180/12/6	180	12	6
180/6/6	180	6	6
180/6/3	180	6	3
190/12/6	190	12	6
190/6/6	190	6	6
190/6/3	190	6	3

**Table 2:** Chemical composition of paulownia wood.

Component	Content (%)
Holocellulose	77.91 ± 0.44
Cellulose	43.25 ± 0.10
Hemicelluloses	34.66 ± 0.23
Pentosans	21.60 ± 0.68
Lignin	25.52 ± 0.40
Substances extracted in ethanol	11.51 ± 0.07
Ash	0.68 ± 0.02

**Table 3:** Determination of weight percentage gain, density, and bulking coefficient.

Modification Variant	*WPG* (%)	Density (g/cm^3^)	*BC* (%)
control	–	0.26 (0.04)	–
180/12/6	−4.86 (1.13)	0.25 (0.04)	−0.06 (0.59)
180/6/6	−1.01 (0.53)	0.26 (0.04)	−1.48 (1.48)
180/6/3	−3.23 (1.01)	0.25 (0.04)	−0.06 (0.59)
190/12/6	−5.70 (1.22)	0.24 (0.03)	−0.15 (1.17)
190/6/6	−2.28 (0.79)	0.25 (0.04)	−1.17 (0.98)
190/6/3	−4.46 (1.16)	0.24 (0.04)	−0.57 (1.13)

**Table 4:** The characteristic bands in the FTIR spectra of *Paulownia elongata* wood.

Wavenumber [cm^−1^]	Assignment/Functional Group	Ref.
3337	O–H stretching in phenolic and aliphatic structures	[34,35,36,37]
2880	C–H stretching in aromatic methoxyl groups and methyl and methylene groups of side chains	[34,35,36,37,38]
1733	C=O stretching in unconjugated ketone/aldehyde	[34,36,37,38,39]
1595	C=C stretching of the aromatic ring; C=O stretching	[36,38,39]
1506	C=C stretching of the aromatic ring	[34,36,38,39]
1430	C–H deformation in –CH_3_, –CH_2_	[34,36,37,38,39]
1370	C–H deformation	[34,36,38,39]
1325	C–O of aromatic ring	[34,35,36,37,38,39]
1240	C-O stretching and O–H in plane in polysaccharides	[34,36,37,38,39]
1154	C–O–C asymmetric stretching in polysaccharides	[36,38,39]
1030	C–O, C=C and C–C–O stretching	[36,38]
897	C-H deformation in cellulose	[39]

**Table 5:** Color parameters of paulownia wood after thermal modification.

Modification Variant	Color Coordinates				
	L*	a*	b*	c*	*h*
Control	77.58 (±3.33)	4.11 (±0.55)	17.22 (±1.23)	17.65 (±1.13)	76.54 (±1.51)
180/6/6	66.13 (±4.91)	6.40 (±1.03)	21.74 (±0.93)	22.54 (±0.85)	73.53 (±2.37)
180/6/3	50.89 (±5.79)	8.78 (±0.77)	21.01 (±1.59)	22.78 (±1.60)	67.29 (±1.86)
180/12/6	49.09 (±4.16)	9.13 (±0.35)	19.77 (±1.24)	21.79 (±1.12)	64.95 (±1.78)
190/6/6	58.75 (±4.08)	7.36 (±0.78)	21.66 (±2.02)	23.04 (±1.92)	70.86 (±1.76)
190/6/3	53.24 (±3.44)	8.51 (±0.37)	20.30 (±0.92)	22.02 (±0.88)	67.23 (±1.20)
190/12/6	43.94 (±3.96)	8.57 (±0.46)	17.62 (±2.22)	19.60 (±2.16)	63.86 (±2.35)

**Table 6:** Color changes of paulownia wood after thermal modification.

Modification Variant	Color Coordinates					
	ΔL*	Δa*	Δb*	Δc*	Δ*h*	Δ*E*
180/6/6	−13.87 (±3.90)	2.90 (±0.62)	4.43 (±1.07)	5.04 (±1.07)	−1.04 (±1.45)	14.96 (±3.62)
180/6/3	−18.69(±20.60)	4.93 (±1.09)	5.62 (±2.20	6.82 (±2.36	−3.06 (±0.59)	23.13 (±4.73)
180/12/6	−29.10 (±2.09)	5.35 (±0.89)	3.22 (±2.23)	4.77 (±2.28)	−4.05 (±0.60)	29.87 (±5.16)
190/6/6	−19.94 (±5.29)	3.65 (±0.89)	3.91 (±2.23)	4.78 (±2.28)	−2.42 (±0.60)	20.68 (±5.16)
190/6/3	−26.64 (±4.13)	4.79 (±0.36)	3.00 (±1.06)	4.34 (±0.99)	−3.17 (±1.93)	27.31 (±4.09)
190/12/6	−33.71 (±20.14)	4.81 (±0.48)	1.22 (±1.43)	2.23 (±1.41)	−4.31 (±0.42)	34.10 (±3.85)

**Table 7:** Descriptive statistics of ultimate stress in compression parallel to the fibers.

Compressive Strength (MPa)							
	Control	180/12/6	180/6/6	180/6/3	190/12/6	190/6/6	190/6/3
Mean	21.2	24.54	26.93	25.47	24.67	24.88	24.16
Minimum	18.8	17.96	20.08	16.66	21.27	18.06	18.60
Maximum	25.51	30.15	33.19	32.59	28.18	31.18	30.89
SD	1.95	4.95	4.40	4.67	2.23	4.24	3.80

**Table 8:** Two-way ANOVA results.

Source	Sum of Squares	Degrees of Freedom (df)	F Value	*p*-Value
Temp. (180 vs. 190 °C)	26.750238	1	1.428049	0.239897
Steam Dosing Time (3 h vs. 6 h)	12.953854	1	0.691535	0.411125
Temp: Time	1.758544	1	0.093879	0.761068
Residual	674.352624	36	–	–

F—value of the F-test statistic; *p*—significance level.

## Data Availability

The raw data supporting the conclusions of this article will be made available by the authors upon request (due to upon request due to agreements with third-party collaborators.).
